# Diacetyl odor shortens longevity conferred by food deprivation in *C.* *elegans* via downregulation of DAF‐16/FOXO

**DOI:** 10.1111/acel.13300

**Published:** 2020-12-31

**Authors:** Sangsoon Park, Murat Artan, Dae‐Eun Jeong, Hae‐Eun H. Park, Heehwa G. Son, Sieun S. Kim, Yoonji Jung, Yunji Choi, Jin I. Lee, Kyuhyung Kim, Seung‐Jae V. Lee

**Affiliations:** ^1^ Department of Life Sciences Pohang University of Science and Technology Pohang South Korea; ^2^ Department of Biological Sciences Korea Advanced Institute of Science and Technology Daejeon South Korea; ^3^ Division of Biological Science and Technology College of Science and Technology Yonsei University Wonju South Korea; ^4^ Department of Brain and Cognitive Sciences Daegu Gyeongbuk Institute of Science and Technology Daegu South Korea

**Keywords:** aging, *C. elegans*, diacetyl, dietary restriction, longevity

## Abstract

Dietary restriction extends lifespan in various organisms by reducing the levels of both nutrients and non‐nutritional food‐derived cues. However, the identity of specific food‐derived chemical cues that alter lifespan remains unclear. Here, we identified several volatile attractants that decreased the longevity on food deprivation, a dietary restriction regimen in *Caenorhabditis elegans*. In particular, we found that the odor of diacetyl decreased the activity of DAF‐16/FOXO, a life‐extending transcription factor acting downstream of insulin/IGF‐1 signaling. We then demonstrated that the odor of lactic acid bacteria, which produce diacetyl, reduced the nuclear accumulation of DAF‐16/FOXO. Unexpectedly, we showed that the odor of diacetyl decreased longevity independently of two established diacetyl receptors, ODR‐10 and SRI‐14, in sensory neurons. Thus, diacetyl, a food‐derived odorant, may shorten food deprivation‐induced longevity via decreasing the activity of DAF‐16/FOXO through binding to unidentified receptors.

## Graphical abstract

Food deprivation, a dietary restriction regimen in *C. elegans*, promotes longevity by increasing nuclear localization and transcriptional activity of DAF‐16/FOXO. The odor of diacetyl, a volatile organic compound that is produced by lactic acid bacteria, decreases food deprivation‐mediated longevity by downregulating DAF‐16/FOXO via an unidentified receptor. Our data indicate that specific food odors can reduce longevity in animals.

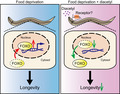

## 1 INTRODUCTION, RESULTS, DISCUSSION

Dietary restriction (DR) promotes longevity by reducing nutrients and restricting access to food‐derived cues. In *Drosophila melanogaster*, odorants derived from live yeast decrease longevity conferred by DR (Libert et al., 2007). In *Caenorhabditis elegans*, food‐derived soluble cues shorten longevity conferred by food deprivation (FD) (Smith et al., 2008), a DR regimen that completely removes both nutrients and chemical cues (Kaeberlein et al., 2006; Lee et al., 2006; Steinkraus et al., 2008; Sutphin & Kaeberlein, 2008), via downregulating DAF‐16/FOXO signaling (Artan et al., 2016). However, the identity of the specific food‐derived chemical cues that can alter aging and lifespan remains unknown.

**FIGURE 1:**
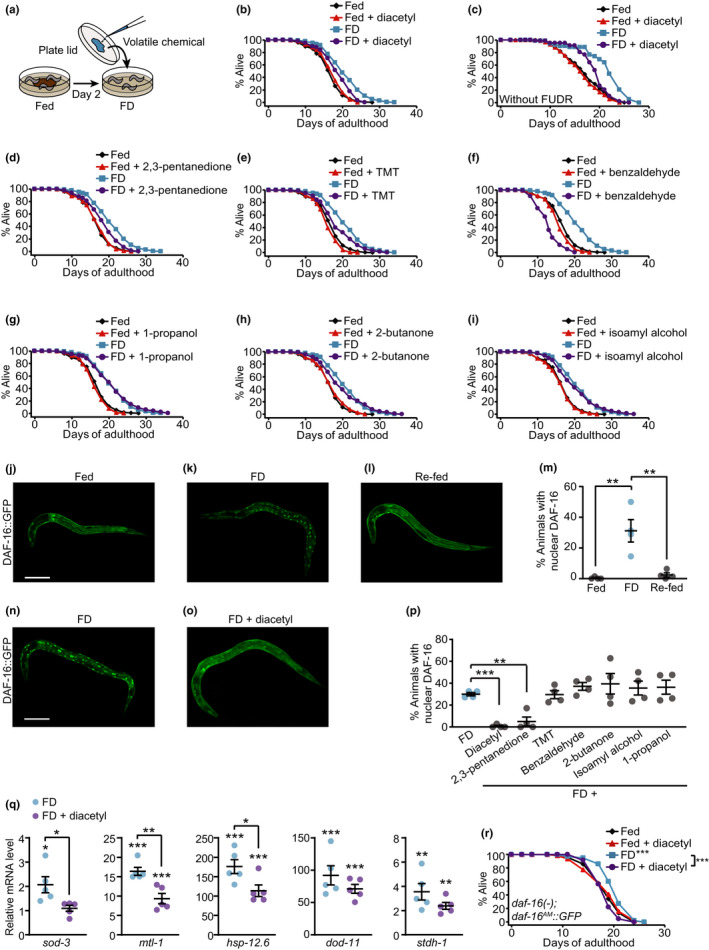
The odor of diacetyl decreases longevity conferred by food deprivation via downregulating DAF‐16/FOXO. (a) Experimental scheme of lifespan assays with volatile chemicals. Fed day 2 adult worms were transferred to plates without bacteria (food deprivation: FD) with each of the volatile chemicals placed on the back of the plate lid. (b‐i) The effect of diacetyl (b, c), 2,3‐pentanedione (d), 2,4,5‐trimethylthiazole (TMT) (e), benzaldehyde (f), 1‐propanol (g), 2‐butanone (h), or isoamyl alcohol (i, see Supporting Discussion) on the lifespan of fed and FD animals. The odor of TMT, 2‐butanone, or isoamyl alcohol reduced FD‐mediated longevity in one out of two independent replicates (Table S1). The effect of diacetyl on lifespan without FUDR treatment (c, see note in Experimental Procedures). Lifespan curves for the chemical screen were obtained by pooling two independent experiments, but the statistical analysis of individual lifespan data is included in Table S1. (j‐l) Images of *daf‐16::GFP* transgenic worms under fed (j), FD (k), or FD followed by re‐feeding with OP50 (re‐fed, l). (m) Increased nuclear localization of DAF‐16::GFP by FD was suppressed in re‐fed conditions (N = 4, > 512 animals per condition). (n, o) Images of *daf‐16::GFP* transgenic worms under FD (n) and FD with diacetyl (o). Scale bar: 50 µm. (p) The quantification of the effects of specific odors on the nuclear localization of DAF‐16::GFP in animals under FD (N = 4, > 100 animals per condition). (q) Expression changes of five selected DAF‐16 target genes, *sod‐3*, *mtl‐1*, *hsp‐12.6*, *dod‐11*, and *stdh‐1*, by FD and the odor of diacetyl (N = 5, *p* values were calculated against fed conditions [Figure S1]). *p < 0.05, **p < 0.01, ***p < 0.001, two‐tailed Student's *t* test. Error bar: standard error of mean. (r) The life‐shortening effects of diacetyl odor on *daf‐16(mu86)*; *daf‐16^AM^*::*GFP* [*daf‐16(‐); daf‐16^AM^::GFP*] worms under FD. See Table S1 for statistical analysis of the lifespan data

Here we sought to identify bacteria‐derived volatile chemicals that act as signaling molecules to modulate lifespan. We first tested whether *C. elegans* lifespan was altered by any of seven volatile organic compounds that have been established as attractants (Bargmann, 2006; Bargmann et al., 1993; Sengupta et al., 1996): diacetyl, 2,3‐pentanedione, 2,4,5‐trimethylthiazole (TMT), benzaldehyde, 1‐propanol, 2‐butanone, and isoamyl alcohol. We exposed animals under FD to each of these seven volatile chemicals and measured lifespan (Figure 1a). Importantly, the chemoattractants diacetyl, 2,3‐pentanedione, TMT, and benzaldehyde substantially reduced the longevity induced by FD (Figure 1b‐f). In contrast, exposure to 1‐propanol, 2‐butanone, or isoamyl alcohol did not affect lifespan (Figure 1g‐i).

We then tested whether these same seven volatile chemicals affected the nuclear localization of DAF‐16/FOXO, which was increased upon FD and linked to longevity (Artan et al., 2016; Fletcher & Kim, 2017; Henderson & Johnson, 2001) (Figure 1j‐m). Exposure to diacetyl or 2,3‐pentanedione substantially decreased the level of nuclear DAF‐16::GFP (Figure 1n‐p). In contrast, the odor of the other volatile chemicals, including TMT and benzaldehyde that reduced longevity conferred by FD, did not (Figure 1p). We concluded that diacetyl and 2,3‐pentanedione may shorten longevity conferred by FD by decreasing the activity of DAF‐16/FOXO.

We focused our analysis on diacetyl, which displayed the greatest effect on the subcellular localization of DAF‐16/FOXO (Figure 1n‐p), and reduced FD‐mediated longevity (Figure 1b,c). We determined whether the odor of diacetyl affected the transcriptional activity of DAF‐16/FOXO by using qRT‐PCR. Among five selected DAF‐16 target genes upregulated by FD, the mRNA levels of *sod‐3*, *mtl‐1*, and *hsp‐12.6* were substantially reduced by the odor of diacetyl, whereas decreases in those of *dod‐11* and *stdh‐1* were not significant (Figure 1q and Figure S1). The odor of diacetyl also suppressed FD‐induced longevity in *daf‐16(‐); daf‐16^AM^::GFP* worms (Figure 1r), which express constitutively nuclear DAF‐16/FOXO (Lin et al., 2001). These data suggest that diacetyl decreases FD‐mediated longevity by downregulating DAF‐16/FOXO via decreasing the transcriptional activity as well as its nuclear localization.

**FIGURE 2:**
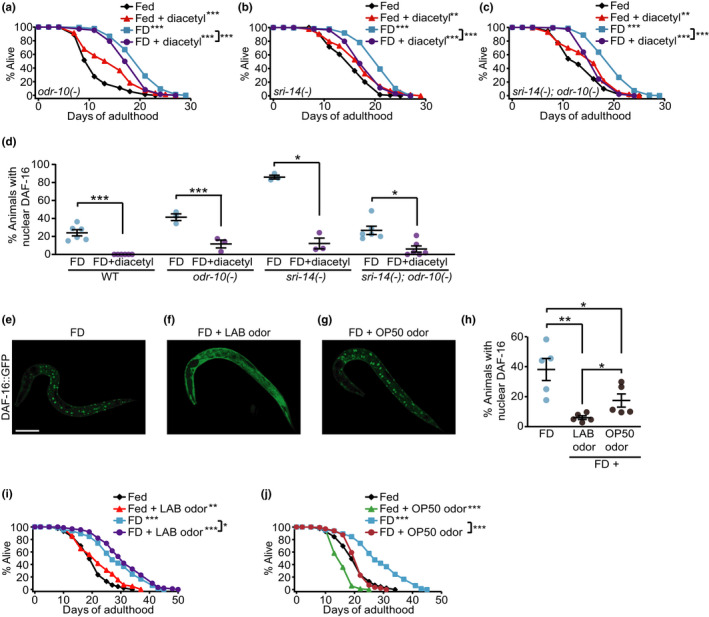
Diacetyl reduces longevity by food deprivation through unidentified receptors and lactic acid bacteria odor downregulates DAF‐16/FOXO. (a‐c) The life‐shortening effects of diacetyl on *odr‐10(ky225)* [*odr‐10(‐)*] (a), *sri‐14(ok2865)* [*sri‐14(‐)*] (b), or *sri‐14(‐)*; *odr‐10(‐)* (c) mutant worms under food deprivation (FD). (d) The effect of diacetyl on the nuclear localization of DAF‐16::GFP under FD in wild‐type (WT) (N = 6), *odr‐10(‐)* (N = 3), *sri‐14(‐)* (N = 3), and *sri‐14(‐)*; *odr‐10(‐)* (N = 6) worms (> 90 animals per condition). (e‐g) Images of *daf‐16::GFP* transgenic worms under FD (e) and FD with the odor of lactic acid bacteria (LAB) *L. paracasei* (f) or OP50 *E. coli* (g). (h) The quantification of data shown in (e‐g) (N = 5, > 891 animals per condition). *p < 0.05, **p < 0.01, ***p < 0.001, two‐tailed Student's *t* test. (i, j) The effects of LAB or OP50 odor on the lifespan of worms under fed and FD conditions. See Table S1 for statistical analysis of the lifespan data

Next, we asked whether diacetyl decreased lifespan or DAF‐16/FOXO activity via acting through its known chemosensory receptors, ODR‐10 and SRI‐14 (Sengupta et al., 1996; Taniguchi et al., 2014). Unexpectedly, mutations in *odr‐10* and/or *sri‐14* did not abrogate the suppressive effect of diacetyl on longevity (Figure 2a‐c) or the nuclear localization of DAF‐16/FOXO upon FD (Figure 2d). These data suggest that diacetyl decreases lifespan and DAF‐16/FOXO activity through unidentified chemical receptors (see Supporting Discussion).

We then sought to determine whether diacetyl produced under physiological conditions affected the activity of DAF‐16/FOXO. We exposed *C. elegans* to the odor of lactic acid bacteria (LAB), *Lactobacillus paracasei*, which produce diacetyl (Choi et al., 2016), and subsequently determined the subcellular localization of DAF‐16/FOXO. We found that the odor of diacetyl‐producing *L*. *paracasei* substantially reduced the nuclear localization of DAF‐16/FOXO (Figure 2e,f,h). In contrast, the odor of *E. coli* OP50 marginally reduced the nuclear localization of DAF‐16/FOXO (Figure 2g,h). We tested whether the odor of LAB or OP50 suppressed the longevity conferred by FD, but did not observe specific suppression (Figure 2i,j). These data suggest that diacetyl‐producing LAB odor downregulates DAF‐16/FOXO but is insufficient to alter longevity under FD (see Supporting Discussion).

Specific food‐derived cues that modulate longevity conferred by FD remained unknown. Here, we showed that diacetyl produced by LAB, a potential diet of *C. elegans* in nature, decreased the activity of DAF‐16/FOXO, a longevity‐promoting transcription factor acting downstream of insulin/IGF‐1 signaling. We also found that the odor of diacetyl shortened FD‐induced longevity. Food odor has been shown to trigger metabolic and physiological changes in *Drosophila* (Lushchak et al., 2015), mice (Brandt et al., 2018), and humans (Smeets et al., 2010). Thus, it will be interesting to determine whether specific food odors can affect longevity in other organisms, including mammals.

## CONFLICT OF INTEREST

The authors declare no competing interests.

## AUTHOR CONTRIBUTIONS

SP, MA, KK, and SJVL designed the study. SP, MA, DEJ, HEHP, HGS, SSK, and YJJ performed experiments. SP, MA, and SJVL analyzed the data. YC and JIL contributed to designing experiments using *L. paracasei*. SP and SJVL wrote manuscript. KK and SJVL supervised the study.

## Supporting information

Supporting Information

## Data Availability

The data that support the findings of this study are available in the Supporting Material of this article.
