# Supplementary material for: Diacetyl odor shortens longevity conferred by food deprivation in C. elegans via downregulation of DAF‐16/FOXO
Source: Aging Cell. 2020 Dec 31;20(1):e13300. doi: 10.1111/acel.13300 (PMC7811839; doi:10.1111/acel.13300)
Supplement: Supplementary file 1 — Supporting Information [file ACEL-20-e13300-s001.pdf]

Figure S1

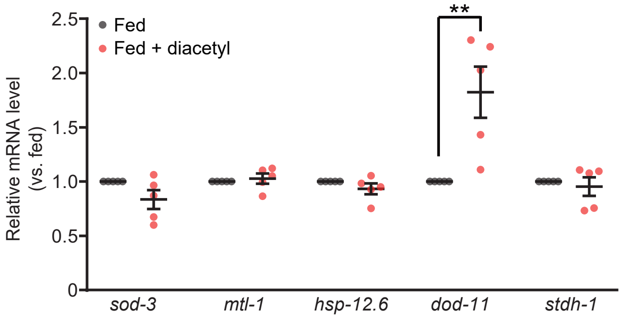

Fed
Fed + diacetyl
**
Relative mRNA level (vs. fed)
2.5
2.0
1.5
1.0
0.5
0
sod-3
mtl-1
hsp-12.6
dod-11
stdh-1

Supporting Information

Supporting Figure Legends

Figure S1. The odor of diacetyl has small effects on the expression of five selected DAF-16/FOXO target genes under fed conditions. qRT-PCR analysis for the expression changes of five selected DAF-16 target genes, *sod-3*, *mtl-1*, *hsp-12.6*, *dod-11*, and *stdh-1*, by the odor of diacetyl in fed conditions (N=5, **$P < 0.01$, two-tailed Student's *t*-test). Error bar: standard error of mean.

Supporting Discussion

It remains unclear whether the lack of diacetyl odor in diets extends lifespan

Despite showing the sufficiency of diacetyl for reducing food deprivation (FD)-mediated longevity, we were not able to test whether the absence of diacetyl odor extends lifespan. That is because we used *E. coli*, which do not produce diacetyl (Chuang and Collins, 1968), as *C. elegans* diets. It will be intriguing to determine whether the absence of diacetyl extends lifespan by comparing the effects of diacetyl-producing wild-type lactic acid bacteria (LAB) and mutant LAB that do not produce diacetyl on FD-mediated longevity in future studies.

Various chemical odors differentially affect worm lifespan

Here, we showed that different volatile chemicals displayed differential effects on the subcellular localization of DAF-16/FOXO and adult lifespan. For example, 2,3-pentanedione shortened longevity under FD and partly decreased the nuclear localization of DAF-16/FOXO, although to a lesser extent than diacetyl. TMT and benzaldehyde also shortened the lifespan of food-deprived animals, but did not affect the subcellular localization of DAF-16/FOXO. Therefore, it seems likely that different olfactory cues modulate lifespan via distinct mechanisms. Different from our results (Figure 1i), the odor of isoamyl alcohol has been shown to extend lifespan (Kurino, Furuhashi, Sudoh, & Sakamoto, 2017). The previous study used water as solvent for diluting isoamyl alcohol, whereas we used ethanol, following the method of a previous paper (Bargmann et al., 1993). Therefore, these differences in experimental conditions may underlie the difference in lifespan results. Elucidating different molecular mechanisms by which unique volatile chemical cues modulate lifespan will be important to understand how *C. elegans* alter physiological states in response to environmental changes.

The odor of diacetyl may affect lifespan via known or unknown chemical receptors

Our negative results using *odr-10* and/or *sri-14* mutants for the effects of diacetyl on the DAF-16 subcellular localization and longevity under FD raise the possibility that diacetyl acts independently of ODR-10 and SRI-14 for regulating these processes. However, it

remains possible that experimental conditions that we used were not exhaustive to exclude the role of ODR-10 and SRI-14. Specifically, the diacetyl concentration that we used (1%) may not have been optimal to determine the role of the diacetyl receptors in longevity or DAF-16/FOXO localization. Indeed, *odr-10* and *sri-14* mutants still display chemotaxis to 1% diacetyl (Taniguchi et al., 2014). Therefore, different concentrations of diacetyl (e.g. 0.1%), to which chemotaxis is substantially impaired in *odr-10* mutants (Taniguchi et al., 2014), may help determining whether the effect of diacetyl on the localization of DAF-16/FOXO and longevity is mediated at least partially by ODR-10.

Another possible scenario is that diacetyl receptors other than ODR-10 and SRI-14 may mediate the effects of diacetyl on lifespan. Chemical cues can elicit long-term changes in development by modulating endocrine signaling and gene expression, or acute behavioral changes via regulating sensory responses. These differential responses upon perception of the same chemical may be mediated by distinct receptors. For example, *C. elegans* ascaroside pheromones regulate long-term dauer formation and short-term avoidance responses via distinct sets of receptors (Kim et al., 2009; Jang et al., 2012). Thus, it seems possible that the effects of diacetyl on the regulation of DAF-16/FOXO activity and longevity under FD conditions are regulated by receptors distinct from ODR-10 and SRI-14 that mediate acute chemotaxis behaviors.

The concentration of diacetyl in LAB may not have been sufficient for affecting longevity under FD

Our finding showing that the odor of LAB, which reduced nuclear localization of DAF-16, did not suppress the longevity conferred by FD was unexpected. We speculate that the odor of diacetyl produced by LAB in our experimental conditions was weaker and lasted for a shorter time than that of pure diacetyl. Our data are consistent with this possibility, because the effect of LAB odor on the nuclear localization of DAF-16 was weaker than that of pure diacetyl (Figure 1p and 2d vs. Figure 2h). It will be important to test whether the odor of highly concentrated LAB reduces the lifespan of worms under FD in future research.

The odor of diacetyl may act as a food signal that affects worm physiology

Diacetyl is produced by LAB, which are abundant in rotting fruits where *C. elegans* reside and often attract the animals (Choi et al., 2016). Thus, *C. elegans* may detect diacetyl as a food signal, and modulate physiology to metabolize food instantaneously upon ingestion. This event may in turn promote growth and reproduction over maintenance of functions that contribute to longevity conferred by FD. This is consistent with our findings that diacetyl increased the lifespan of fed worms 7 out of 9 trials but reduced that of worms under FD (Table S1). We speculate that the odor of diacetyl acts as a signal for the presence of foods, inducing physiological responses that support body function for longevity under food-enriched conditions. In contrast, a false nutrient signal elicited by the odor of diacetyl may exhaust resources under nutrient-depleted conditions, leading to decreased lifespan. We were not able to experimentally test this

possibility because determining the developmental rate and the fecundity of *C. elegans* with the odor of LAB or diacetyl without food is not technically feasible, because worms need nutrients for growth and reproduction. Therefore, developing tasteless and odorless *C. elegans* diets, if possible, will be crucial for testing this important question.

Experimental Procedures

Strains

C. elegans strains were cultured on nematode growth medium (NGM) plates seeded with *E. coli* OP50 at 20ºC. Some of the strains used in this study were obtained from Caenorhabditis Genetics Center, which is funded by the NIH Office of Research Infrastructure (P40 OD010440). The *C. elegans* strains used in this study are as follows: wild-type Bristol strain N2, TJ356 *zIs356[daf-16p::daf-16a/b::gfp; rol-6]*, IJ1370 *odr-10(ky225)* outcrossed four times to N2, IJ1565 *sri-14(ok2865)* outcrossed four times to N2, IJ1617 *sri-14(ok2865); odr-10(ky225)*, IJ1656 *zIs356[daf-16p::daf-16::gfp; rol-6]; odr-10(ky225)*, IJ1655 *sri-14(ok2865); zIs356[daf-16p::daf-16::gfp; rol-6]*, IJ1657 *sri-14(ok2865); zIs356[daf-16p::daf-16::gfp; rol-6]; odr-10(ky225)*, CF2747 *daf-16(mu86); muIs113[daf-16p::GFP::daf-16^{AM}; rol-6D]*. *Lactobacillus paracasei* (KCTC 3510) used in this research was obtained from Korean Collection for Type Cultures (KCTC), South Korea.

Lifespan assays

Lifespan assays were performed as previously described (Artan et al., 2016), with some modifications. Briefly, age-synchronized *C. elegans* larvae were cultured on OP50-seeded NGM plates until the worms reached pre-fertile young adult stage (day 0) at 20ºC. The worms were then transferred to NGM plates containing 5 μM 5-fluoro-2'-deoxyuridine (FUDR, Sigma-Aldrich, MO, USA) to prevent progeny from hatching [Note: we used FUDR unless otherwise noted because a large fraction of animals displayed internal hatching under FD (Tables S2 and S3). In addition, all the papers that reported lifespan extension by food deprivation (FD) in *C. elegans* used FUDR or 5-fluorouracil to prevent progeny from hatching (Kaeberlein et al., 2006; Lee et al., 2006; Smith et al., 2008; Steinkraus et al., 2008; Sutphin & Kaeberlein, 2008; Mehta et al., 2009; Schleit, Wall, Simko, & Kaeberlein, 2011; Sutphin, Bishop, Yanos, Moller, & Kaeberlein, 2012; Cabreiro et al., 2013; Leiser, Fletcher, Begun, & Kaeberlein, 2013; Thondamal, Witting, Schmitt-Kopplin, & Aguilaniu, 2014; Chandler-Brown et al., 2015; Artan et al., 2016; Choi et al., 2019; Buis et al., 2019).]. After two days, worms were transferred to either OP50-seeded NGM plates for fed conditions or NGM plates containing 50 μg/ml ampicillin (USB, Santa Clara, CA, USA), to prevent bacterial growth, for FD. Worms were counted as dead when they did not respond to a gentle touch using a platinum wire. Worms that crawled off the plates, ruptured, displayed internal hatching, or burrowed were censored but included in the statistical analysis. Lifespan assays with FUDR treatment were performed by at least two independent researchers for reproducibility and analyzed individually. Lifespan assays without FUDR treatment were

performed by at least two independent researchers and pooled for statistical analysis, because of the extremely small number of animals (1 to 35) after censoring for each trial. Statistical analysis was performed by using a log-rank (Mantel–Cox method) test in OASIS (online application of survival analysis, https://sbi.postech.ac.kr/oasis) (Yang et al., 2011) and OASIS2 (https://sbi.postech.ac.kr/oasis2) (Han et al., 2016).

Odor treatment

To expose worms with volatile chemical odors, 10 µl of each volatile chemical (1%) dissolved in ethanol was spread on the back of the lid of each NGM plate and the plate was immediately closed with the lid. Volatile chemicals used in this study: diacetyl (2,3-butanedione; Sigma-Aldrich, MO, USA), 2,3-pentanedione (Sigma-Aldrich, MO, USA), 2,4,5-trimethylthiazole (TMT; Sigma-Aldrich, MO, USA), benzaldehyde (Sigma-Aldrich, MO, USA), 1-propanol (Sigma-Aldrich, MO, USA), 2-butanone (Sigma-Aldrich, MO, USA), and isoamyl alcohol (3-methylbutanol; Sigma-Aldrich, MO, USA). Ethanol was used as a solvent control following the method described previously (Bargmann et al., 1993). To expose worms with bacterial odor, each block (36 mm^3 and 144 mm^3, respectively, for DAF-16::GFP subcellular localization and lifespan assays) of LB agar media with overnight culture of OP50 or citrate agar media with 48 h culture of *L. paracasei* was placed on the back of the lid of each NGM plate. The composition of citrate agar media was based on a previous report (Choi, Yoon, Subbammal Kalichamy, Yoon, & Lee, 2016); 5 g yeast extract (BD, Belgium), 0.1 g $MgSO_4 \cdot 7H_2O$ (Sigma-

Aldrich, MO, USA), 0.05 g $MnSO_4 \cdot H_2O$ (Sigma-Aldrich, MO, USA), 2 g NaH_2PO_4 (Sigma-Aldrich, MO, USA), 2.2 g $HOC(COONa)(CH_2COONa)_2 \cdot 2H_2O$ (Sigma-Aldrich, MO, USA), 6.3 g D(+)-glucose (Junsei Chemical, Japan), 1 ml Tween 80 (Sigma-Aldrich, MO, USA), and 15 g agar (Duchefa Biochemie, Netherlands) were dissolved in 1 L double-distilled water (ddH_2O). Control agar media (15 g agar and 5 g yeast extract dissolved in 1 L ddH_2O) was used as a control for bacterial odor lifespan assays. Each of the odor-treated plates was enclosed in different plastic bags for DAF-16::GFP subcellular localization assays or plastic boxes for lifespan assays to prevent cross-contamination by volatile chemicals. For lifespan assays, worms were exposed to the odor of each volatile chemical or a bacteria-cultured agar block every day until the lifespan assays were completed.

Measurement of DAF-16::GFP subcellular localization

To determine the subcellular localization of DAF-16::GFP, L2 or L3 stages of *daf-16::gfp* transgenic animals were used as previously reported (Artan et al., 2016) with minor modifications; L2 or L3 larvae were used because autofluorescence and growing germline in adults hindered the visual inspection of DAF-16::GFP localization in the intestinal cells. Synchronized L2 or L3 stage *daf-16::gfp* transgenic animals were collected from NGM agar plates seeded with *E*. *coli* OP50 by using M9 buffer. The worms were then washed four times with M9 buffer to remove residual bacteria, and were placed on ampicillin (50 µg/ml)-containing NGM agar plates without bacteria for 24

h for FD experiments. The worms under FD were exposed to the odor of volatile chemicals or bacteria for 30 min. The number of worms displaying nuclear-localized DAF-16::GFP was counted using fluorescence microscopes (Stereo Discovery V8 or HRc Zeiss Axioscope A.1, Carl Zeiss, Germany). Worms that exhibited green fluorescence in the nuclei of the intestinal cells and clearly distinguishable nuclear outlines were considered as worms with nuclear-localized DAF-16::GFP. A fixation method of worms was employed to preserve the images of DAF-16::GFP in experimental conditions. Briefly, the worms were harvested and washed twice with ddH_2O. One ml of 4% paraformaldehyde was added to the worms, which were subsequently nutated for 45 min at room temperature. After a brief spin down using a table-top centrifuge, the fixed worms were washed twice with phosphate-buffered saline (PBS, pH 7.4, Thermo Fisher Scientific, Waltham, MA, USA) and stored in 70% ethanol before imaging.

Microscopy

Fixed *daf-16p::daf-16::gfp* worms were mounted on a 2% agarose pad on a slide glass. Confocal microscopy images were captured by using an inverted LSM 880 laser scanning microscope (Carl Zeiss, Germany) with Plan-Apochromat 20X 0.8 M27 objective. Excitation wavelength at 488 nm and emission wavelength at 526 nm were used to detect green fluorescence.

qRT-PCR analysis

Synchronized L4 or pre-fertile young adult animals grown at 20ºC were treated with 50 μM FUDR (Sigma-Aldrich, MO, USA) to prevent progeny from hatching. At day two adulthood, worms were harvested with M9 buffer, washed three times with M9 buffer, and placed on OP50-seeded NGM plates (fed) or NGM plates containing 50 μg/ml ampicillin (USB, Santa Clara, CA, USA) (FD) treated with FUDR. After 24 h, the worms were exposed to ethanol (control) or diacetyl for 6 h. The worms were then harvested with M9 buffer and used for RNA extraction and qRT-PCR analysis. Total RNA was extracted by using RNAiso plus (Takara, Japan), and ImProm-II Reverse Transcriptase (Promega, WI, USA) was used to synthesize cDNA from the total RNA. qPCR was performed with *Power* SYBR Green PCR master mix (Applied Biosystems, Thermo Fisher Scientific, Waltham, MA, USA) using StepOne real-time PCR system (Applied Biosystems, Thermo Fisher Scientific, Waltham, MA, USA). Comparative C_T method was used for quantitative analysis of target gene expression via StepOne software (version 2.3). *pmp-3* mRNA level was used as a normalization control. Following primers were used for qRT-PCR.

pmp-3

Forward: GTTCCCGTGTTCATCACTCAT

Reverse: ACACCGTCGAGAAGCTGTAGA

sod-3

Forward: CTATCTTCTGGACCAACTTGG

Reverse: GCAAGTTATCCAGGGAACCG

mtl-1

Forward: GACTGCTGAAATTAAGAAATCATG

Reverse: GTCTCCACTGCATTCACATTTGTC

hsp-12.6

Forward: GGAGTTGTCAATGTCCTCGACG

Reverse: GAAGTTCTCCAATGTTCTTGAC

dod-11

Forward: GAACAAGCTGTTGAATACGTCAG

Reverse: GAAAGTAACCTTTGAATCCTTTG

stdh-1

Forward: CGTCAATATTGGATCAGTTGC

Reverse: CAACGTATTTCTTGGTAGCAG

Supporting Tables

Table S1. Lifespan data analysis.

**Strain/treatment**	**Mean lifespan ± s.e.m. (days)**	**75th percentile**	**% lifespan change**	**Number of animals that died/total**	***P* value vs. control**	**Figure in text**
N2/fed control	16.5±0.4	20		121/150		Fig. 1b, d-i
N2/fed diacetyl	16.6±0.4	20	0.9%	117/150	0.7682	Fig. 1b
N2/fed 2,3-pentanedione	16.9±0.4	20	2.6%	112/150	0.5620	Fig. 1d
N2/fed TMT	15.9±0.3	18	-3.4%	123/150	0.0756	Fig. 1e
N2/fed benzaldehyde	16.1±0.3	18	-2.3%	121/150	0.2083	Fig. 1f
N2/fed 1-propanol	16.0±0.4	18	-2.8%	102/150	0.1723	Fig. 1g

N2/fed 2-butanone	16.8±0.4	20	1.9%	117/150	0.5699	Fig. 1h
N2/fed isoamyl alcohol	16.3±0.4	20	-1.0%	123/150	0.7709	Fig. 1i
N2/FD control	21.1±0.6	24	27.8%[Fed]	95/150	<0.0001[Fed]	Fig. 1b, d-i
N2/FD diacetyl	18.3±0.5	22	-13.1% (11.1%[Fed])	96/150	0.0001 (<0.001[Fed])	Fig. 1b
N2/FD 2,3-pentanedione	17.8±0.5	22	-15.4% (8.1%[Fed])	92/150	<0.0001 (<0.001[Fed])	Fig. 1d
N2/FD TMT	19.9±0.6	24	-5.3% (21.1%[Fed])	99/150	0.2241 (<0.0001[Fed])	Fig. 1e
N2/FD benzaldehyde	13.2±0.3	16	-37.1% (-19.7%[Fed])	85/150	<0.0001 (<0.0001[Fed])	Fig. 1f
N2/FD 1-propanol	21.7±0.7	26	2.9% (31.5%[Fed])	97/150	0.1852 (<0.0001[Fed])	Fig. 1g
N2/FD 2-butanone	21.0±0.7	26	-0.4%	100/150	0.5553	Fig. 1h

			(27.3%[Fed])		(<0.0001[Fed])	
N2/FD isoamyl alcohol	21.5±0.6	26	2.3% (30.7%[Fed])	102/150	0.3382 (<0.0001[Fed])	Fig. 1i
N2/fed control	17.2±0.3	18		103/120		Fig. 1b, d-i
N2/fed diacetyl	18.3±0.3	20	6.8%	87/120	<0.05	Fig. 1b
N2/fed 2,3-pentanedione	16.9±0.3	20	-1.5%	107/120	0.8466	Fig. 1d
N2/fed TMT	16.2±0.3	18	-5.5%	96/120	<0.01	Fig. 1e
N2/fed benzaldehyde	15.6±0.3	18	-9.1%	106/120	<0.001	Fig. 1f
N2/fed 1-propanol	16.7±0.3	18	-2.5%	103/120	0.1835	Fig. 1g
N2/fed 2-butanone	17.5±0.4	20	1.9%	91/120	0.3972	Fig. 1h

N2/fed isoamyl alcohol	16.7±0.3	18	-2.9%	104/120	0.2877	Fig. 1i
N2/FD control	20.6±0.4	24	20.3%[Fed]	69/120	<0.0001[Fed]	Fig. 1b, d-i
N2/FD diacetyl	19.6±0.3	22	-4.8% (14.5%[Fed])	73/120	<0.05 (<0.0001[Fed])	Fig. 1b
N2/FD 2,3-pentanedione	19.6±0.3	22	-5.1% (14.1%[Fed])	82/120	<0.05 (<0.0001[Fed])	Fig. 1d
N2/FD TMT	17.3±0.3	18	-16.3% (0.7%[Fed])	82/120	<0.0001 (0.9888[Fed])	Fig. 1e
N2/FD benzaldehyde	12.7±0.4	13	-38.5% (-26.0%[Fed])	67/120	<0.0001 (<0.0001[Fed])	Fig. 1f
N2/FD 1-propanol	20.0±0.4	22	-2.9% (16.9%[Fed])	72/120	0.2891 (<0.0001[Fed])	Fig. 1g
N2/FD 2-butanone	18.4±0.4	20	-10.9% (7.2%[Fed])	68/120	0.0001 (<0.05[Fed])	Fig. 1h

N2/FD isoamyl alcohol	18.9±0.4	22	-8.2% (10.4%[Fed])	83/120	<0.01 (<0.001[Fed])	Fig. 1i
N2/fed control*	16.9±0.3	20		208/330		Fig. 1c
N2/fed diacetyl*	16.4±0.3	19	-2.8%	242/360	0.1633	Fig. 1c
N2/FD control*	21.5±0.7	24	26.9%[Fed]	42/330	<0.0001[Fed]	Fig. 1c
N2/FD diacetyl*	19.0±0.5	21	-11.3% (12.6%[Fed])	49/360	0.0001 (<0.05[Fed])	Fig. 1c
N2/fed control*	15.3±0.2	17		127/300		
N2/fed diacetyl*	15.8±0.3	17	3.5%	151/300	0.1096	
N2/FD control*	23.7±2.1	27	54.8%[Fed]	7/800	<0.0001[Fed]	
N2/FD diacetyl*	20.7±2.8	27	-12.8% (35.1%[Fed])	3/800	0.5222 (0.0126[Fed])	
N2/fed control	14.4±0.3	16		111/150		
N2/fed diacetyl	17.5±0.4	22	21.1%	120/150	<0.0001	
N2/FD control	19.4±0.3	22	34.7%[Fed]	122/200	<0.0001[Fed]	
N2/FD diacetyl	16.7±0.2	18	-14.0%	164/200	<0.0001	

			(15.9%^{Fed})		(<0.0001^{Fed})	
daf-16(-); daf-16^{AM}*::GFP*/<br>fed control	18.1±0.3	21	25.1%^{N2}	129/150	<0.0001^{N2}	
daf-16(-); daf-16^{AM}*::GFP*/<br>fed diacetyl	18.5±0.3	21	2.6%	135/150	0.3552	
daf-16(-); daf-16^{AM}*::GFP*/<br>FD control	20.5±0.2	23	13.7%^{Fed}	172/200	<0.0001^{Fed}	
daf-16(-); daf-16^{AM}*::GFP*/<br>FD diacetyl	17.9±0.2	19	-13.0%<br>(-1.0%^{Fed})	184/200	<0.0001<br>(<0.05^{Fed})	
N2/fed control	15.1±0.3	18		147/150		
N2/fed diacetyl	19.4±0.4	25	28.5%	144/150	<0.0001	
N2/FD control	21.4±0.3	25	39.2%^{Fed}	163/200	<0.0001^{Fed}	
N2/FD diacetyl	17.8±0.2	20	-15.4%<br>(17.7%^{Fed})	176/200	<0.0001<br>(<0.0001^{Fed})	

daf-16(-); daf-16^{AM}::GFP/ fed control	18.7±0.3	21	23.4%^{N2}	120/120	<0.0001^{N2}	Fig. 1r
daf-16(-); daf-16^{AM}::GFP/ fed diacetyl	18.6±0.4	21	-0.4%	120/120	0.5853	Fig. 1r
daf-16(-); daf-16^{AM}::GFP/ FD control	20.5±0.3	24	10.0%^{Fed}	150/150	<0.0001^{Fed}	Fig. 1r
daf-16(-); daf-16^{AM}::GFP/ FD diacetyl	18.4±0.2	19	-10.1% (-1.1%^{Fed})	150/150	<0.0001 (0.0776^{Fed})	Fig. 1r
N2/fed control	18.2±0.3	22		141/150		
N2/fed diacetyl	22.5±0.4	25	23.5%	140/150	<0.0001	
N2/FD control	19.4±0.4	22	6.1%^{Fed}	107/200	<0.05^{Fed}	
N2/FD diacetyl	16.0±0.3	18	-17.3% (-12.2%^{Fed})	101/200	<0.0001 (<0.0001^{Fed})	
daf-16(-); daf-16^{AM}::GFP/ fed control	18.9±0.3	21	3.3%^{N2}	133/150	0.7481^{N2}	

daf-16(-); daf-16^{AM}::GFP/ fed diacetyl	20.1±0.4	24	6.8%	138/150	<0.001	
daf-16(-); daf-16^{AM}::GFP/ FD control	20.2±0.3	21	7.0%^{Fed}	128/200	<0.001^{Fed}	
daf-16(-); daf-16^{AM}::GFP/ FD diacetyl	17.3±0.2	19	-14.1% (-8.1%^{Fed})	139/200	<0.0001 (<0.0001^{Fed})	
N2/fed control	13.6±0.3	16		157/180		
N2/fed diacetyl	16.1±0.4	20	18.7%	158/180	<0.0001	
N2/FD control	16.4±0.2	19	21.0%^{Fed}	163/210	<0.0001^{Fed}	
N2/FD diacetyl	15.2±0.2	17	-7.1% (12.4%^{Fed})	141/210	<0.0001 (<0.0001^{Fed})	
odr-10(ky225)/fed control	12.3±0.2	13	-9.1%^{N2}	161/180	<0.001^{N2}	
odr-10(ky225)/fed diacetyl	15.5±0.3	18	25.8%	158/180	<0.0001	

odr-10(ky225)/FD control	16.7±0.2	19	35.6%[Fed]	181/210	<0.0001[Fed]	
odr-10(ky225)/FD diacetyl	15.2±0.2	17	-9.0% (23.5%[Fed])	155/210	<0.0001 (<0.0001[Fed])	
sri-14(ok2865)/fed control	15.3±0.3	18	12.8%[N2]	159/180	<0.0001[N2]	
sri-14(ok2865)/fed diacetyl	18.9±0.3	22	23.3%	151/180	<0.0001	
sri-14(ok2865)/FD control	18.5±0.2	21	20.9%[Fed]	162/210	<0.0001[Fed]	
sri-14(ok2865)/FD diacetyl	16.1±0.2	17	-13.0% (5.1%[Fed])	137/210	<0.0001 (0.1651[Fed])	

sri-14(ok2865); odr-10(ky225)/fed control	12.7±0.2	16	-6.1%[N2]	159/180	<0.05[N2]	
sri-14(ok2865); odr-10(ky225)/fed diacetyl	16.7±0.3	20	31.4%	157/180	<0.0001	
sri-14(ok2865); odr-10(ky225)/FD control	16.3±0.3	19	27.7%[Fed]	106/175	<0.0001[Fed]	
sri-14(ok2865); odr-10(ky225)/FD diacetyl	16.3±0.2	17	0.1% (27.7%[Fed])	138/210	0.7412 (0.0001[Fed])	
N2/fed control	13.6±0.3	16		151/180		
N2/fed diacetyl	15.5±0.4	18	14.1%	151/180	<0.0001	
N2/FD control	20.3±0.3	24	49.6%[Fed]	151/175	<0.0001[Fed]	

N2/FD diacetyl	17.6±0.3	21	-13.2% (29.9%^{Fed})	103/210	<0.0001 (<0.0001^{Fed})	
odr-10(ky225)/fed control	11.3±0.3	13	-16.8%^{N2}	166/180	<0.0001^{N2}	Fig. 2a
odr-10(ky225)/fed diacetyl	14.4±0.4	18	27.1%	161/180	<0.0001	Fig. 2a
odr-10(ky225)/FD control	20.4±0.3	24	80.3%^{Fed}	184/210	<0.0001^{Fed}	Fig. 2a
odr-10(ky225)/FD diacetyl	18.5±0.3	21	-9.4% (63.4%^{Fed})	139/210	<0.0001 (<0.0001^{Fed})	Fig. 2a
sri-14(ok2865)/fed control	15.5±0.4	18	13.9%^{N2}	134/180	<0.001^{N2}	Fig. 2b
sri-14(ok2865)/fed diacetyl	17.1±0.4	21	10.8%	140/180	<0.001	Fig. 2b

sri-14(ok2865)/FD control	21.3±0.3	24	37.6%^{Fed}	181/210	<0.0001^{Fed}	Fig. 2b
sri-14(ok2865)/FD diacetyl	18.6±0.3	21	-12.7% (20.1%^{Fed})	178/210	<0.0001 (<0.0001^{Fed})	Fig. 2b
sri-14(ok2865); odr-10(ky225)/fed control	13.8±0.3	16	1.4%^{N2}	167/180	0.6259^{N2}	Fig. 2c
sri-14(ok2865); odr-10(ky225)/fed diacetyl	15.4±0.4	18	12.1%	159/180	<0.001	Fig. 2c
sri-14(ok2865); odr-10(ky225)/FD control	20.2±0.3	24	46.8%^{Fed}	138/175	<0.0001^{Fed}	Fig. 2c

sri-14(ok2865); odr-10(ky225)/FD diacetyl	16.9±0.2	17	-16.5% (22.6%[Fed])	184/210	<0.0001 (<0.0001[Fed])	Fig. 2c
N2/fed control	16.3±0.3	19		116/120		
N2/fed diacetyl	17.0±0.3	19	4.1%	115/120	<0.01	
N2/FD control	21.2±0.3	23	29.9%[Fed]	106/200	<0.0001[Fed]	
N2/FD diacetyl	17.9±0.2	20	-15.9% (9.2%[Fed])	121/200	<0.0001 (<0.0001[Fed])	
sri-14(ok2865); odr-10(ky225)/fed control	13.7±0.3	17	-16.0%[N2]	111/120	<0.0001[N2]	
sri-14(ok2865); odr-10(ky225)/fed diacetyl	15.8±0.3	19	15.3%	115/120	<0.0001	
sri-14(ok2865); odr-10(ky225)/FD	21.9±0.3	23	59.9%[Fed]	103/185	<0.0001[Fed]	

control						
sri-14(ok2865); odr-10(ky225)/FD diacetyl	19.2±0.2	20	-12.5% (39.9%^{Fed})	107/200	<0.0001 (<0.0001^{Fed})	
N2/fed control	17.7±0.4	20		109/120		
N2/fed diacetyl	17.8±0.4	22	0.6%	118/120	0.3739	
N2/FD control	23.5±0.3	24	32.3%^{Fed}	117/200	<0.0001^{Fed}	
N2/FD diacetyl	20.5±0.2	21	-12.4% (15.8%^{Fed})	97/200	<0.0001 (<0.0001^{Fed})	
sri-14(ok2865); odr-10(ky225)/fed control	16.6±0.3	20	-6.3%^{N2}	111/120	<0.01^{N2}	
sri-14(ok2865); odr-10(ky225)/fed diacetyl	16.8±0.4	20	1.3%	103/120	0.2702	

sri-14(ok2865); odr-10(ky225)/FD control	24.0±0.2	24	44.3%[Fed]	99/200	<0.0001[Fed]	
sri-14(ok2865); odr-10(ky225)/FD diacetyl	21.6±0.2	24	-9.9% (29.9%[Fed])	112/200	<0.0001 (<0.0001[Fed])	
N2/fed agar control	20.1±0.6	22		96/150		Fig. 2i,j
N2/fed LAB odor	22.1±0.7	27	10.1%	109/150	<0.01	Fig. 2i
N2/fed OP50 odor	15.9±0.3	19	-21.0%	105/150	<0.0001	Fig. 2j
N2/FD agar control	28.7±0.8	34	42.6%[Fed]	101/200	<0.0001[Fed]	Fig. 2i,j
N2/FD LAB odor	31.3±0.7	37	9.0% (41.2%[LAB])	117/200	<0.05 (<0.0001[LAB])	Fig. 2i
N2/FD OP50 odor	21.1±0.4	22	-26.5%	112/200	<0.0001	Fig. 2j

			(32.7%OP50)		(<0.0001OP50)	
N2/fed agar control	20.4±0.5	23		110/150		
N2/fed LAB odor	21.6±0.6	25	5.9%	101/120	<0.05	
N2/fed OP50 odor	16.3±0.3	18	-20.0%	123/150	<0.0001	
N2/FD agar control	24.7±0.7	28	21.4%Fed	73/120	<0.0001Fed	
N2/FD LAB odor	25.5±0.8	32	3.1% (18.2%LAB)	77/120	0.1119 (<0.0001LAB)	
N2/FD OP50 odor	21.7±0.3	25	-12.1% (33.4%OP50)	131/200	<0.0001 (<0.0001OP50)	
N2/fed agar control	18.2±0.3	20		101/150		
N2/fed LAB odor	22.0±0.5	26	20.6%	124/150	<0.0001	
N2/fed OP50 odor	16.9±0.2	18	-7.1%	110/150	<0.0001	

N2/FD agar control	25.4±0.5	28	39.1%^{Fed}	77/150	<0.0001^{Fed}	
N2/FD LAB odor	27.6±0.6	30	8.8% (25.4%^{LAB})	69/150	<0.001 (<0.0001^{LAB})	
N2/FD OP50 odor	22.4±0.3	23	-11.7% (32.2%$^{\mathrm{OP50}}$)	83/150	<0.0001 (<0.0001$^{\mathrm{OP50}}$)	

Lifespan data within the double-solid lines are biological replicates from the same experimental sets, and lifespan data within single-solid lines were performed in parallel and statistical analysis was performed within the sets. Different conditions within a single set were distinguished by dotted lines. Worms under food deprivation were designated as FD. All percent lifespan changes and *P* values for odor-treated conditions were calculated against control conditions within each fed or FD groups with the same genotype otherwise noted with superscripts described below. Lifespan data marked with asterisk (*) were performed without FUDR treatment, and were pooled from two independent biological replicates within the same experimental sets because of an extremely high censoring rate caused by FD (Table S2 and Table S3). *P* values were calculated using Mantel-Cox log-rank test.

^{Fed}: percent lifespan change or *P* value against fed control animals with same genotype.

[N2]: percent lifespan change or *P* value against fed control N2 within the same experimental set.

[LAB]: percent lifespan change or *P* value against LAB odor-treated fed control N2 within the same experimental set.

[OP50]: percent lifespan change or *P* value against OP50 odor-treated fed control N2 within the same experimental set.

Table S2. Analysis of internal hatching by food deprivation in lifespan assays performed with FUDR treatment.

Strain/treatment	Number of animals with internal hatching	Number of total censored animals	Number of total animals	% internal hatching	% Censored	Related figure in text
N2/fed control	0	29	150	0%	19.3%	Fig. 1b
N2/fed diacetyl	0	33	150	0%	22.0%	Fig. 1b
N2/FD control	0	55	150	0%	36.7%	Fig. 1b

N2/FD diacetyl	0	54	150	0%	36.0%	Fig. 1b
N2/fed control	0	17	120	0%	14.2%	Fig. 1b
N2/fed diacetyl	0	33	120	0%	27.5%	Fig. 1b
N2/FD control	0	51	120	0%	42.5%	Fig. 1b
N2/FD diacetyl	0	47	120	0%	39.2%	Fig. 1b
N2/fed control	0	39	150	0%	26.0%	Fig. 1r
N2/fed diacetyl	0	30	150	0%	20.0%	Fig. 1r
N2/FD control	0	78	200	0%	39.0%	Fig. 1r
N2/FD diacetyl	0	36	200	0%	18.0%	Fig. 1r
N2/fed control	0	3	150	0%	2.0%	Fig. 1r
N2/fed diacetyl	0	6	150	0%	4.0%	Fig. 1r
N2/FD control	0	37	200	0%	18.5%	Fig. 1r
N2/FD diacetyl	0	24	200	0%	12.0%	Fig. 1r
N2/fed control	0	9	150	0%	6.0%	Fig. 1r
N2/fed diacetyl	0	10	150	0%	6.7%	Fig. 1r

N2/FD control	0	93	200	0%	46.5%	Fig. 1r
N2/FD diacetyl	0	99	200	0%	49.5%	Fig. 1r
N2/fed control	0	23	180	0%	12.8%	Fig. 2a-c
N2/fed diacetyl	0	22	180	0%	12.2%	Fig. 2a-c
N2/FD control	0	47	210	0%	22.4%	Fig. 2a-c
N2/FD diacetyl	0	69	210	0%	32.9%	Fig. 2a-c
N2/fed control	0	29	180	0%	16.1%	Fig. 2a-c
N2/fed diacetyl	0	29	180	0%	16.1%	Fig. 2a-c
N2/FD control	0	24	175	0%	13.7%	Fig. 2a-c
N2/FD diacetyl	0	107	210	0%	51.0%	Fig. 2a-c

N2/fed control	0	4	120	0%	3.3%	Fig. 2c
N2/fed diacetyl	0	5	120	0%	4.2%	Fig. 2c
N2/FD control	0	94	200	0%	47.0%	Fig. 2c
N2/FD diacetyl	0	79	200	0%	39.5%	Fig. 2c
N2/fed control	0	11	120	0%	9.2%	Fig. 2c
N2/fed diacetyl	0	2	120	0%	1.7%	Fig. 2c
N2/FD control	0	83	200	0%	41.5%	Fig. 2c
N2/FD diacetyl	0	103	200	0%	51.5%	Fig. 2c

Internal hatching and censoring rates were calculated from the lifespan data that were performed with FUDR treatment. Different experimental sets are separated by double-solid lines, and biological replicates from the same experimental sets are distinguished by single-solid lines. Different conditions within a single experiment were distinguished by dotted lines. Worms under food deprivation were designated as FD.

Table S3. Analysis of internal hatching caused by food deprivation in lifespan assays performed without FUDR treatment.

Strain/treatment	Number of animals with internal hatching	Number of total censored animals	Number of total animals	% internal hatching	% Censored	Related figure in text
N2/fed control*	79	122	330	23.9%	37.0%	Fig. 1c
N2/fed diacetyl*	77	118	360	21.4%	32.8%	Fig. 1c
N2/FD control*	240	288	330	72.7%	87.3%	Fig. 1c
N2/FD diacetyl*	264	311	360	73.3%	86.4%	Fig. 1c
N2/fed control*	141	172	300	47.0%	57.3%	Fig. 1c
N2/fed diacetyl*	124	149	300	41.3%	49.7%	Fig. 1c
N2/FD control*	565	793	800	70.6%	99.1%	Fig. 1c
N2/FD diacetyl*	625	797	800	78.1%	99.6%	Fig. 1c

Internal hatching and censoring rates were calculated from analyzing the lifespan datasets marked with asterisk (*) in Table S1, which were performed without FUDR treatment. Different experimental sets are separated by double-solid lines, and different conditions within a single experiment were distinguished by dotted lines. Worms under

food-deprived conditions were designated as FD. Worms displayed severe internal hatching phenotype during lifespan assays without FUDR treatment, and the internal hatching rate was substantially increased by FD; we observed no internal hatching phenotype while performing lifespan assays with FUDR treatment (Table S2). The odor of diacetyl did not affect the internal hatching rate of worms under either fed or food-deprived conditions without FUDR. Therefore, our data showing that diacetyl reduced the lifespan of worms under FD with or without FUDR treatment (Figure 1b, c) suggest that internal hatching does not seem to affect the lifespan of fed or food-deprived worms treated with the odor of diacetyl.

Supporting References

Artan, M., Jeong, D. E., Lee, D., Kim, Y. I., Son, H. G., Husain, Z., . . . Lee, S.-J. V. (2016). Food-derived sensory cues modulate longevity via distinct neuroendocrine insulin-like peptides. *Genes Dev, 30*(9), 1047-1057. doi:10.1101/gad.279448.116

Bargmann, C. I., Hartwieg, E., & Horvitz, H. R. (1993). Odorant-selective genes and neurons mediate olfaction in *C. elegans*. *Cell, 74*(3), 515-527. doi:10.1016/0092-8674(93)80053-h

Buis, A., Bellemin, S., Goudeau, J., Monnier, L., Loiseau, N., Guillou, H., & Aguilaniu, H. (2019). Coelomocytes Regulate Starvation-Induced Fat Catabolism and Lifespan Extension through the Lipase LIPL-5 in *Caenorhabditis elegans*. Cell Rep, 28(4), 1041-

1049.e1044. doi:10.1016/j.celrep.2019.06.064

Cabreiro, F., Au, C., Leung, K. Y., Vergara-Irigaray, N., Cochemé, H. M., Noori, T., . . . Gems, D. (2013). Metformin retards aging in *C. elegans* by altering microbial folate and methionine metabolism. Cell, 153(1), 228-239. doi:10.1016/j.cell.2013.02.035

Chandler-Brown, D., Choi, H., Park, S., Ocampo, B. R., Chen, S., Le, A., . . . Kaeberlein, M. (2015). Sorbitol treatment extends lifespan and induces the osmotic stress response in *Caenorhabditis elegans*. Front Genet, 6, 316. doi:10.3389/fgene.2015.00316

Choi, H., Cho, S. C., Ha, Y. W., Ocampo, B., Park, S., Chen, S., . . . Kaeberlein, M. (2019). DDS promotes longevity through a microbiome-mediated starvation signal. Transl Med Aging, 3, 64-69. doi:10.1016/j.tma.2019.07.001

Choi, J. I., Yoon, K. H., Subbammal Kalichamy, S., Yoon, S. S., & Lee, J. I. (2016). A natural odor attraction between lactic acid bacteria and the nematode *Caenorhabditis elegans*. *Isme j, 10*(3), 558-567. doi:10.1038/ismej.2015.134

Chuang, L. F., & Collins, E. B. (1968). Biosynthesis of diacetyl in bacteria and yeast. J Bacteriol, 95(6), 2083-2089. doi:10.1128/jb.95.6.2083-2089.1968

Han, S. K., Lee, D., Lee, H., Kim, D., Son, H. G., Yang, J. S., . . . Kim, S. (2016). OASIS 2: online application for survival analysis 2 with features for the analysis of maximal lifespan and healthspan in aging research. *Oncotarget, 7*(35), 56147-56152. doi:10.18632/oncotarget.11269

Jang, H., Kim, K., Neal, S. J., Macosko, E., Kim, D., Butcher, R. A., . . . Sengupta, P. (2012). Neuromodulatory state and sex specify alternative behaviors through antagonistic synaptic pathways in *C. elegans*. Neuron, 75(4), 585-592. doi:10.1016/j.neuron.2012.06.034

Kaeberlein, T. L., Smith, E. D., Tsuchiya, M., Welton, K. L., Thomas, J. H., Fields, S., . . . Kaeberlein, M. (2006). Lifespan extension in *Caenorhabditis elegans* by complete removal of food. *Aging Cell, 5*(6), 487-494. doi:10.1111/j.1474-9726.2006.00238.x

Kim, K., Sato, K., Shibuya, M., Zeiger, D. M., Butcher, R. A., Ragains, J. R., . . . Sengupta, P. (2009). Two chemoreceptors mediate developmental effects of dauer pheromone in *C. elegans*. Science, 326(5955), 994-998. doi:10.1126/science.1176331

Kurino, C., Furuhashi, T., Sudoh, K., & Sakamoto, K. (2017). Isoamyl alcohol odor promotes longevity and stress tolerance via DAF-16 in *Caenorhabditis elegans*. Biochem Biophys Res Commun, 485(2), 395-399. doi:10.1016/j.bbrc.2017.02.066

Lee, G. D., Wilson, M. A., Zhu, M., Wolkow, C. A., de Cabo, R., Ingram, D. K., & Zou, S. (2006). Dietary deprivation extends lifespan in *Caenorhabditis elegans*. *Aging Cell, 5*(6), 515-524. doi:10.1111/j.1474-9726.2006.00241.x

Leiser, S. F., Fletcher, M., Begun, A., & Kaeberlein, M. (2013). Life-span extension from hypoxia in *Caenorhabditis elegans* requires both HIF-1 and DAF-16 and is antagonized by SKN-1. J Gerontol A Biol Sci Med Sci, 68(10), 1135-1144. doi:10.1093/gerona/glt016

Mehta, R., Steinkraus, K. A., Sutphin, G. L., Ramos, F. J., Shamieh, L. S., Huh, A., . . .

Kaeberlein, M. (2009). Proteasomal regulation of the hypoxic response modulates aging in *C. elegans*. Science, 324(5931), 1196-1198. doi:10.1126/science.1173507

Schleit, J., Wall, V. Z., Simko, M., & Kaeberlein, M. (2011). The MDT-15 subunit of mediator interacts with dietary restriction to modulate longevity and fluoranthene toxicity in *Caenorhabditis elegans*. PLoS One, 6(11), e28036. doi:10.1371/journal.pone.0028036

Smith, E. D., Kaeberlein, T. L., Lydum, B. T., Sager, J., Welton, K. L., Kennedy, B. K., & Kaeberlein, M. (2008). Age- and calorie-independent life span extension from dietary restriction by bacterial deprivation in *Caenorhabditis elegans*. BMC Dev Biol, 8, 49. doi:10.1186/1471-213x-8-49

Steinkraus, K. A., Smith, E. D., Davis, C., Carr, D., Pendergrass, W. R., Sutphin, G. L., . . . Kaeberlein, M. (2008). Dietary restriction suppresses proteotoxicity and enhances longevity by an *hsf-1*-dependent mechanism in *Caenorhabditis elegans*. *Aging Cell, 7*(3), 394-404. doi:10.1111/j.1474-9726.2008.00385.x

Sutphin, G. L., & Kaeberlein, M. (2008). Dietary restriction by bacterial deprivation increases life span in wild-derived nematodes. *Exp Gerontol, 43*(3), 130-135. doi:10.1016/j.exger.2007.10.019

Sutphin, G. L., Bishop, E., Yanos, M. E., Moller, R. M., & Kaeberlein, M. (2012). Caffeine extends life span, improves healthspan, and delays age-associated pathology in *Caenorhabditis elegans*. Longev Healthspan, 1, 9. doi:10.1186/2046-2395-1-9

Thondamal, M., Witting, M., Schmitt-Kopplin, P., & Aguilaniu, H. (2014). Steroid hormone signalling links reproduction to lifespan in dietary-restricted *Caenorhabditis elegans*. Nat Commun, 5, 4879. doi:10.1038/ncomms5879

Yang, J. S., Nam, H. J., Seo, M., Han, S. K., Choi, Y., Nam, H. G., . . . Kim, S. (2011). OASIS: online application for the survival analysis of lifespan assays performed in aging research. *PLoS One, 6*(8), e23525. doi:10.1371/journal.pone.0023525
